# Absence of Coronavirus RNA in Faecal Samples from Wild Primates in Gabon, Central Africa

**DOI:** 10.3390/pathogens12101272

**Published:** 2023-10-23

**Authors:** Illich Manfred Mombo, Océane Rieu, Matthieu Fritz, Larson Boundenga, Telstar Ndong Mebaley, Clark Mbou-Boutambe, Léadisaelle Hosanna Lenguiya, Gael Darren Maganga, Virginie Rougeron, Franck Prugnolle, Fredéric Thomas, Eric M. Leroy

**Affiliations:** 1 Centre International de Recherches Médicales de Franceville, Franceville BP 769, Gabon; boundenga@gmail.com (L.B.); clarkmbou@gmail.com (C.M.-B.); gael_maganga@yahoo.fr (G.D.M.); 2 Institut de Recherche pour le Développement (IRD), Maladies Infectieuses et Vecteurs, Écologie, Génétique, Évolution et Contrôle (MIVEGEC), Université de Montpellier-IRD 224-CNRS 5290, 34394 Montpellier, France; oceane.rieu@ird.fr (O.R.); fritz.matthieu@iage-france.com (M.F.); telstarlunique@gmail.com (T.N.M.); frederic.thomas2@ird.fr (F.T.); eric.leroy@ird.fr (E.M.L.); 3 Faculté des Sciences et Techniques, Université Marien Ngouabi, Brazzaville BP 69, Congo; hosannelenguiya@gmail.com; 4 International Research Laboratory-REHABS, CNRS-Université Lyon 1-Nelson Mandela University, Nelson Mandela University George Campus, George 6531, South Africa; rougeron.virginie@gmail.com (V.R.); prugnolle@gmail.com (F.P.)

**Keywords:** coronaviruses, chimpanzee, gorilla, mandrill, surveillance, zoonosis

## Abstract

Coronaviruses (CoVs, *Coronaviridae*) are a diverse group of viruses that infect mammals, birds, and fish. Seven CoVs infect humans, among which Severe Acute Respiratory Syndrome CoVs-1 and -2 and Middle East respiratory syndrome CoVs have shown how they can impact global health and the economy. Their spillover from bats-the natural reservoir-to humans has required intermediary hosts. Prevention requires that active surveillance be conducted on animals. Today, there is no data concerning the genetic diversity of CoVs naturally circulating in wild primates. This study aimed to screen wild great apes and mandrills in Gabon for CoVs. A total of 229 faecal samples of great apes and mandrills collected from 2009 to 2012 in forests and national parks were used for the detection of CoVs by nested PCR using primers targeting a conserved region of the RNA-dependent RNA polymerase. While all samples were negative, this lack of detection could be related to sample size, the transient nature of the infection, or because faecal samples are not suitable for detecting CoVs in primates. A longitudinal study should be performed and other non-invasive methods used to collect respiratory samples to better evaluate the circulation of CoVs in these primates.

## 1. Introduction

Coronaviruses (CoVs) are a diverse group of viruses belonging to the genera *Alphacoronavirus*, *Betacoronavirus*, *Gammacoronavirus*, and *Deltacoronavirus* of the family *Coronaviridae* (order *Nidovirales*) (https://talk.ictvonline.org/taxonomy/, accessed on 3 December 2022). Alpha-CoVs and beta-CoVs infect mammals, while gamma-CoVs and delta-CoVs infect birds, fish, and sometimes mammals [1]. CoVs are the largest RNA viruses, with genome sizes of 27–32 kb. Because there is a high error rate during replication, the frequency of mutations and recombination is high [2], favouring the possibility of CoVs adapting rapidly to novel hosts and ecological niches [3].

Seven CoVs are known to infect humans: HCoV-229E, HCoV-OC43, HCoV-HKU1, and HCoV-NL63 are responsible for mild symptoms, while Severe Acute Respiratory (SARS)-CoV-1, Middle East Respiratory Syndrome (MERS)-CoV, and SARS-CoV-2 cause severe respiratory diseases and have a higher impact on the global economy and public health. Most of the CoVs infecting humans have a zoonotic origin, and spillover to humans sometimes requires intermediary hosts, i.e., one-humped camels (*Camelus dromedarius*) for MERS-CoV and palm civets (*Paguma larvata*) or common raccoon-dogs (*Nyctereutes procyonoides*) for SARS-CoV-1 [4,5]. While the Malayan pangolin (*Manis javanica*) is thought to be the intermediate host for SARS-CoV-2 [6], this has not yet been confirmed.

The outbreaks of SARS-CoV-1 and 2 and MERS-CoV demonstrate the threats that CoVs pose to human health. The prevention of further outbreaks requires the conduct of active animal surveillance. The interest in testing animals for CoVs increased after the SARS epidemic [7]. Since then, several coronaviruses have been discovered in different species of vertebrate animals [8].

As is the case with all primates worldwide, populations of chimpanzees, gorillas, and mandrills are declining [9]. Although the decline is a major consequence of global changes and human activities, infectious diseases have also contributed [10]. Respiratory diseases caused by pneumoviruses (human metapneumovirus and human respiratory syncytial virus) have caused mortalities in Ivory Coast chimpanzees [11]. Ebolavirus has caused massive mortalities in wild gorillas and chimpanzees in Gabon and the Republic of Congo [12]. Therefore, the search for infectious agents in these animals is doubly advantageous—for assessing primate health and for implementing active surveillance for potentially zoonotic pathogens. Today, there is no data concerning the genetic diversity of CoVs naturally circulating in wild primates. For this study, we took advantage of 229 faecal samples that had already been collected to screen CoVs in wild gorillas (*Gorilla gorilla gorilla*), chimpanzees (*Pan troglodytes troglodytes*), and mandrills (*Mandrillus sphinx*) living in Gabon.

## 2. Materials and Methods

### 2.1. Sample Collection and RNA Extraction

**Figure 1:**
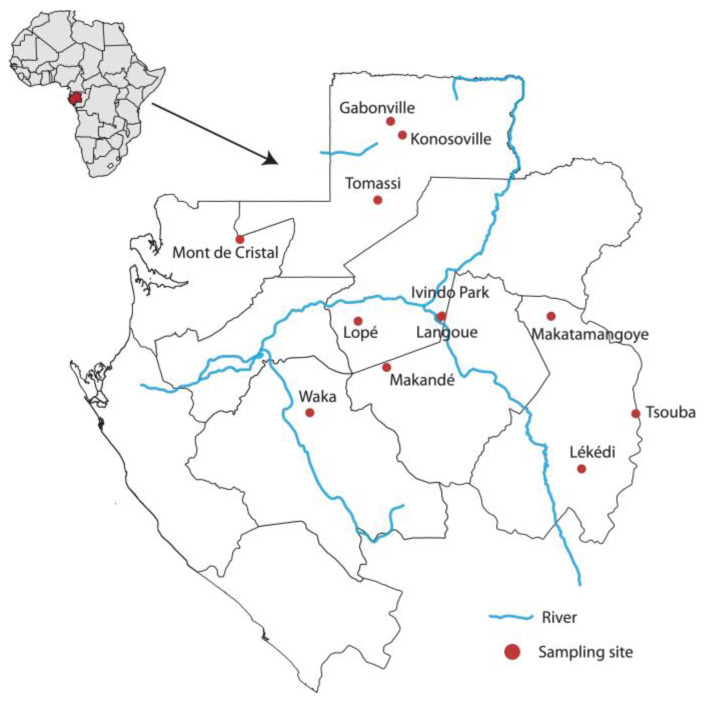
Locations where faecal samples of great apes and mandrills were collected. The image was created using QGIS software version 2.18.15 (https://www.qgis.org/fr/site/, accessed on 12 March 2023).

A total of 229 great apes and mandrill faecal samples that had already been collected for a study on enterovirus diversity and to understand the origin of *Plasmodium* spp. in humans were included in this study [13,14]. These samples were collected between 2009 and 2012 at 13 sites located in national parks and forested areas in Gabon, where contact with humans is minimal (Figure 1). Approximately 20 g of fresh faecal samples were preserved in RNAlater (Ambion) in the field and then stored at −80 °C on arrival at the laboratory. Host species identification of each sample, determined previously, was based on observations in the fields of faeces morphology, vocalisations, night nests, locations where great apes were seen, and molecular methods. Samples were centrifuged at 5000× *g* for 7 min, and RNA was extracted from the supernatant using the Nucleospin RNA Mini kit for RNA purification (Macherey-Nagel, Düren, Germany) according to the manufacturer’s recommendations and stored at −20 °C.

### 2.2. Reverse Transcription and Coronavirus Detection

The extracted RNA was then reverse-transcribed into cDNA using Superscript IV Reverse Transcriptase (Invitrogen, Illkirch, France) in a final volume of 20 μL. Specifically, 10 μL of RNA in a mix consisting of 1 μL dNTPs (10 mM), 1 μL random hexamer, and 1 μL of DNase-free water was incubated at 65 °C for 5 min, followed by 1 min on ice. A second mixture, made up of 4 μL of 5× Superscript IV Buffer, 1 μL of DTT (100 mM), 0.5 μL of RNAse Out (Invitrogen, Carlsbad, CA, USA), 0.5 μL of enzyme, and 1 μL of DNAse-free water, was added to the previous mix. The reverse transcription programme was set to 23 °C for 10 min, 50–55 °C for 10 min, and 80 °C for 10 min. Samples were then screened for CoVs by hemi-nested PCR with a set of primers (5′ Pan-CoV-F1: 5′-GGKTGGGAYTAYCCKAARTG-3′ and Pan-CoV-R1: 3′TGYTGTSWRCARAAYTCRTG-5′; Pan-CoVF2: 5′-GGTTGGGACTATCCTAAGTGTGA-3′, Pan-CoVR2: 3′-CCATCATCAGATAGAATCATCAT-5′) targeting a 440 bp fragment of the RdRp, which is conserved among all known coronaviruses, using Platinum Taq DNA Polymerase (Invitrogen, Carlsbad, CA, USA) [15]. For the first round the mix of 5 μL of cDNA, 2.5 μL of 10× reaction buffer, 0.75 μL of MgCl_2_ (50 mM), 0.5 μL of dNTPs (10 mM), 1 μL of each primer (10 μM) and 0.1 μL of Platinum Taq Enzyme. The second round was performed using 5 μL of the PCR product from the first round, and the mixture included the components and volumes from the first round. Amplification programmes were set up as previously described [15]. SARS-CoV 2 RNA extracted from the nasal swab of a dog that had been tested positive was used as a positive control. Amplicons were visualized in a 1.5% agarose gel after electrophoresis.

### 2.3. Ethic Approval

Research approval for the collection and use of primate faecal samples was obtained from the National Centre of Scientific and Technological Research (CENAREST; permission number AR0031/09).

## 3. Results

Primate species were first identified by observation and then by molecular methods, as previously described [13,14]. The 229 faecal samples used for CoV screening consisted of 87 samples from chimpanzees, 122 from gorillas, and 21 from mandrills. The highest number of samples was obtained in 2012 (136), followed by 2010 (74) and 2009 (19); no samples were collected in 2011. Information on collected samples (date, location) is summarized in Table 1. All extracted RNA from the 229 faecal samples were tested for CoVs by PCR; none were found to be positive for CoVs, while the positive control was evident.

## 4. Discussion

Primates are considered the source of several pathogens that have successfully crossed the species barrier and been transmitted to humans. This study is the first that aims to evaluate the diversity of CoVs in wild great apes and mandrills. Even though all the faecal samples tested were negative, the absence of detection does not allow strong conclusions to be drawn, and the presence of CoVs in wild African great apes and mandrills cannot be ruled out. Indeed, the number of samples tested, the acute nature of the infection, and the viral replication site could explain the absence of detection.

Even though the number of samples is relatively large, it may still not be large enough. Compared to gorilla and chimpanzee samples, the number of mandrill samples was low. In addition, no sampling occurred in 2011, and the samples used were not collected continuously throughout the same year. This could limit the possibility of detecting CoVs that could be seasonal. Contrary to retroviruses, which are responsible for chronic infections, CoV infections are transient. For SARS-CoV 2—probably the most studied coronavirus—the viral RNA in faecal samples could be detected up to three weeks after the onset of symptoms [16]. This feature of transient infection could limit the possibility of shedding.

CoV infection in animals and humans is mainly associated with respiratory and enteric diseases, but it may occasionally affect the reproductive and nervous systems. Some CoVs replicate in the respiratory tract, some in the gastrointestinal tract, and some in both sites; thus, their detection could depend on the type of sample. For example, Enteric bovine CoVs (EBCoV), which cause diarrhoea in calves, were first detected in water buffalo in Bulgaria by virus neutralization and hemagglutination inhibition assays in serum samples [17]. The molecular tool has also been used for the detection of EBCoV in the intestinal contents of water buffalo in Italy [18]. The diagnosis of swine CoVs, such as porcine transmissible gastroenteritis coronavirus (TGEV), porcine epidemic diarrhoea virus (PEDV), and swine acute diarrhoea syndrome virus (SADS-CoV), which infect the gastrointestinal tracts of pigs, uses faecal samples to detect the RNA of these viruses [19]. In contrast, respiratory samples are more suitable for the detection of CoVs that cause respiratory diseases—since replication mainly occurs in the respiratory tract—such as MERS-CoV and HCoV-229E in camelids, Canine Respiratory CoV (CRCoV) in dogs, and SARS-CoV-1 and -2 [20]. The detection of HCoV-OC43 in chimpanzees was performed using a nasal swab. It is possible that CoVs that infect great apes and mandrill replicate in the respiratory system and not in the cells lining the intestines, so a non-invasive sampling strategy needs to be developed to collect respiratory samples in the wild. For example, the nests of apes and mandrills should be inspected and nasal discharge collected along with plants chewed and discarded by primates [21].

Although CoVs were not detected in our study, other experimental studies have shown the ability of these viruses to infect primates. For instance, cynomolgus monkeys (*Macaca fascicularis*), African green monkeys (*Chlorocebus aethiops*), and rhesus macaque (*Macaca mulatta*) have served as animal models for in vivo experiments to understand the pathogenicity and mediated responses of SARS-CoV-1 and MERS-CoV infections [22,23,24]. In addition, HCoV-OC43 has infected wild habituated chimpanzees (*P. t. verus*) and caused respiratory disease in Ivory Coast in 2016 [25].

## 5. Conclusions

In conclusion, the absence of CoVs RNA in faecal samples of great apes and mandrills in Gabon adds no additional information regarding their circulation and diversity in these primates. We suggest that a continuous and longitudinal study be performed to collect and screen a larger number of samples. The development of a non-invasive sampling method and tools to collect respiratory secretions in animal nests would be useful to screen for CoVs that replicate mostly or exclusively in the respiratory system. This could lead to a better evaluation of the genetic diversity and help prevent CoV spillover and spillback to humans. Finally, alternative methods of detection, such as immunological methods and whole-virus protein detection (Western blot, ELISA), may provide more information regarding the passive or active circulation of CoVs in wild primates.

## Figures and Tables

**Table 1:** Number of samples collected by year, the site and primate species.

Year of Collection	Location	Species			Total
		Gorilla	Chimpanzee	Mandrill	
2012	Gabonville	13	_	3	16
	Lyokomillieu	1	_	_	1
	Kokossoville	11	7	_	18
	Langoué	12	1	_	13
	Lopé	7	39	_	46
	Makatamangoye	1	4	_	5
	Tomassi	2	3	17	22
	Waka	10	5	_	15
2010	Lekedi	3	_	_	3
	Lopé	28	13	1	42
	Makande	12	_	_	12
	Mont de Cristal	4	8	_	12
	Parc-Ivindo	1	_	_	1
	Tsouba	_	4	_	4
2009	Lekedi	1	_	_	1
	Lopé	16	2	_	18
Total		122	86	21	229

## Data Availability

The original contributions presented in the study are included in the article. Further inquiries can be directed to the corresponding author.
